# Hydropathy, or the Cold Water Cure, as Practised by Vincent Preissnitz at Graeffenberg, Silesia, Austria

**Published:** 1842-10

**Authors:** 


### Art. IX.

1. *Hydropathy, or the Cold Water Cure, as practised by Vincent Preissnitz at Graeffenberg, Silesia, Austria.* By R. T. Claridge, Esq.—*London*, 1842. 8vo, pp. 318.
2. *A Practical Treatise on the Cure of Diseases by Water, Air, Exercise, and Diet, &c.* By James Wilson, Physician to his Serene Highness the Prince of Nassau, &c.—*London*, 1842. 8vo, pp. 202.

An Austrian peasant among the mountains of Silesia, an unlettered uneducated hind,—in spite of difficulties of situation, remoteness, rudeness, rough roads, and rougher accommodation, and without availing himself of secrecy in his remedy, but on the contrary proclaiming that he uses simple water only,—has induced seven or eight thousand invalids in the course of the last ten years to submit themselves for weeks and months to his treatment; to endure the coarse food of his table d'hôte, the straw mattresses, and barrack-room deprivations of his own dwelling (which is a great hospital), or the still coarser appliances of his mountain village; and to pursue the self-denying routine that he absolutely enjoins, and the discomforts of his applications. He has become notorious throughout Europe, and the publication of the second thousand of Mr. Claridge's work shows that the English public are not likely to be ignorant of his existence. To set down, without inquiry, such a man as a quack, to attribute his reputation and success altogether to the large gullibility of mankind, may be the flattering unction employed by self-love to soothe its less successful efforts, or by indolence to avoid the labour of thought, or by contempt to magnify its own littleness; but is it not rather wiser to admit that such a man must have some ability, that public opinion is not totally wrong or contemptible, that his means must be often successful, and that even "practical men" may learn something from him? For where is there ever a Great Error even, without some truth at the bottom?

The success of such irregular men brings home to us the imperfection of our more regular methods of treating disease. It gives us a glimpse of the region which is still to be opened for discovery; of that ocean of truth lying beyond the shore on which we are now picking up our few shells. For if we could cure all diseases with facility and certainty, *secundum artem*, removing the internal cause of suffering, or restoring the lost power, with the same sureness that the dentist removes the aching tooth, no Austrian peasant would seduce crowds of invalids to visit his rude abode and his wild mountains.

But we cannot do this; and why?

In the first place our art is limited. There are incurable diseases, and there are certain stages at which all diseases are incurable; and as far as books can teach us we are yet deficient in precise rules to guide us as to what diseases and what stages of disease are curable by medicine; to what extent many are curable; and how many may be left to the unaided powers of nature. From this ignorance the practitioner often attempts what is impossible, by subjecting the body to the action of drugs to cure a disease which is beyond their control; or, on the other hand, he neglects remedies from an erroneous belief in their inefficacy; whilst the patient, who is deeply conscious of his own loss of health, after having subjected himself to every routine plan, often flies to the quack or to the pretender who is the most liberal in his promises.

It is easy to see and to point out defects; but the difficulty lies in ascertaining the remedy. The most striking deficiency in our art is in prognosis (foreknowledge). It cannot be denied, even by the most adhesive "laudator temporis acti," that in diagnosis we have made great progress during the last half century. The works of Corvisart, Laennec, Baillie, Abercrombie, Louis, and Bright sufficiently attest this. But can the same be said of prognosis? Are clear laws, or rules, or maxims, or aphorisms yet made out in sufficient number to enable a man, with any certainty, to foretel the progress of a particular case of disease? And can books teach this? Can such fixed maxims be ever laid down so as to be applicable to diseases which differ in every individual? To a certain extent certainly; but within narrow limits. For prognosis is that branch of medicine which more than any other requires not merely experience but long-continued thoughtful and watchful attention to disease. Whilst experience gives to a reflecting practitioner daily additional reasons for feeling humility for his own deficiencies, for the fallacy of his judgment, the treacherousness of his memory, the want of observation of and attention to minute particulars, and for the little power he really possesses over many diseases; it also affords him satisfaction by enabling him to decide with greater certainty as to what cases are likely to be benefited by medicines, and under what circumstances the "nimia diligentia" is even more injurious than complete neglect. The young, the ardent, and the slightly experienced are too much inclined to believe that their power over disease by medicines is unlimited. With all the skill in the diagnosis of organic diseases (often at an incurable stage) which they have acquired at hospitals, with the knowledge of the principles of medicine which they have learned in books or in lecture-rooms, and of the actions of drugs which they have in like manner studied until their insight seems so clear that all difficulties are over, they have to learn other lessons by the sober certainties of actual practice. The belief of the young practitioner in himself, as well as in his remedies, will be much mitigated when he has been baffled day by day by a slight gastrodynia or a trifling headach; rendered anxious by an obstinate and blemishing eruption of acne on a lady's face; or had to listen week after week to the stale story of hypochondriacal distress, alike exhausting to his patience, his sympathies, and his materia medica. More strongly still will he feel his weakness, after having been compelled to watch the slow growth and the unrelenting progress of an abdominal tumour; or the devastation of a malignant fever prostrating and destroying those whose life seems to those around them of the highest value, whilst all his medicaments are at least powerless, if they be not actually injurious. Some men, indeed, there are, of such buoyant and sanguine temperament, of such incurable elasticity, as not to be depressed, dismayed, nor even taught by any such lessons, how often soever repeated, but whose faith in drugs and in themselves reaches into old age. But there are others, on the contrary, as easily discouraged by failure, and who are apt to sink into entire scepticism. Both extremes, like all extremes, are wrong. The practitioner who has gained real knowledge from his experience holds the middle path, or endeavours to keep as near to it as he can, neither disbelieving in the power of medicines, nor credulously trusting in their unfailing efficacy.

One reason that books do not furnish us with all the exact information we require as to what diseases and what stages of disease are incurable and what diseases may be left to nature, is the infinite varieties of forms which even a disease to which we attach a single and well-recognized name takes, according to the causes which produced it, the constitution of the individual, its complication with other diseases, or with morbid peculiarities of organization, and the treatment or external and internal influences to which the patient is subjected. In the great class of febrile complaints, which are more regular than any other, the observation of Sydenham is still applicable, that the treatment (and consequently the prognosis) must vary with the nature of the epidemic, and that the physician must discover for himself (and often by losing several of his first patients), the peculiar means to be adopted for the new form of disease: and what is true of an epidemic is especially applicable to each individual case which differs in some degree from every other of the same species. The most carefully compiled statistical tables can no more furnish satisfactory information as to the duration of a particular instance of disease, as to the recovery or death of the patient, than can the best calculated insurance tables inform us how many years each insured individual has to live. The single case is to be decided on its own individual merits; it may be *the* exception to the most general rule.

There is another difficulty, partly the effect of circumstances, partly of our own creating, which prevents the attainment of more certain knowledge on various other obscure points of medical science.

It need hardly be said that the practice of medicine is in a very artificial and complicated condition in this country; and that from the quantity of drugs taken on all occasions, the powers of nature must be so constantly interfered with that our opportunities of understanding her real power are but few. The *quid pro quo* system, by which the general practitioner has been in the habit of having his services remunerated in this commercial country, is the root of a wide-spread evil. It is a practice alike prejudicial to the progress of medical science, to the health of the patient, and to the reputation of our profession. If it were not so universal a custom, it would be difficult to believe that the English were, on the whole, best satisfied to pay for medical advice by the strange fiction of an extravagant sum charged for the medicines. We by no means wish to assert that on this account patients are purposely compelled to swallow what their medical attendants know will be injurious to them; on the contrary, we believe that mere placebos are constantly prescribed; and that where under other circumstances soda-water, imperial drink, or hot and cold water would be recommended, under the present system, effervescing draughts, saline mixtures, lotions in bottles, and ticketed fomentations are substituted. But still the tendency of our customs is to lead the patient to take and expect, and the medical practitioner to prescribe and to believe in, the efficacy of much more medicine, in many more cases, than is necessary, and to give to mere drugs an importance beyond their due. How many patients believe that little is doing for them unless they have a regular supply of physic, "twice or three times daily," or "every four hours;" and how many practitioners think that when they have prescribed the dose they have done their whole duty? Doctors are too apt to forget that even in cases where "inactivity" in the administration of active remedies is injurious, activity for the patient's welfare may be shown in the perfect remembrance of all his symptoms, and in minute attention to air, rest, or exercise, diet, hours, and habits, and to those other more common circumstances which influence so strongly his comfort and his well-being. This branch of medicine, which the French call hygiène, and for which we have no other term than the Latin word "regimen," is certainly considered of much less importance in the cure of disease than it merits; and undoubtedly a more scrupulous and watchful attention to it, as a branch of medicine second in utility to no other, would be one mode in which the present faulty and artificial state of our medical practice would be improved. Both medical men and their patients would be then on the road to a more moderate estimation of the power of mere drugs in the treatment of diseases. If, indeed, this mode of practice were abandoned the means of our art would be most limited, and our power trifling. For although many diseases may require no active medicines, yet there are none which do not require "regimen," and the regulation of these common influences is as much within the province of medicine as the exhibition of the most powerful drugs. And perhaps more real success, and consequently reputation, attaches to the practice of those who minutely attend to this branch than to those who attach more exclusive importance to the more intricate and apparently deeply scientific parts of medicine. The aphorism of the moralist is here applicable:

> "Verily, methinks
> Wisdom is ofttimes nearer when we stoop
> Than when we soar."

We once knew a provincial practitioner, a man by no means of scientific acquirements, but who was extremely successful in the cure of chronic diseases. In the first place, he minutely inquired into the habits of his patients, to ascertain the causes of their ill health; and in cases in which there was general derangement of the health, of some standing, he adopted one plan, which was this: He sent his patients into the country, ordered them to take exercise on horses, donkeys, or ponies, and on foot, without fatigue, to drink very freely of whey, and to clothe themselves completely with flannel. The medicine he gave (very little) was some mild vegetable tonic pill, to be washed down with chamomile or sage tea. And in this way he cured the nervous, hypochondriacal, obstinately dyspeptic and the debilitated, or improved those incapable of being cured. It may be worth while to add, as examples are more convincing than mere argument, that the most popular provincial physician of the present day is especially attentive to all that relates to the "regimen" of his patients. And to the same cause must be attributed very much of the success which mineral spas deservedly attain; for although the water of each differs, from the purest up to the most highly mineralized, yet at all places "regimen" is much insisted on, and much advantage does arise from the rational way in which the patients are compelled to live; for although the water might not be of much benefit, yet good habits must improve all. In this way also the homœopathists may be of good service, by teaching us how many diseases require no medicine at all, but are (if curable,) to be improved by "regimen" solely.

The foregoing remarks have been suggested by the consideration of the plan (the main subject of this article,) proposing the cure of all diseases which are not in such a stage as to be absolutely incurable, without any medicine whatever, but by the employment of pure, cold water, applied externally and taken internally, together with a close attention to regimen—such as mountain air and constant exposure to it, much exercise, total abstinence from distilled or fermented liquors, and plain and coarse food. To those who would at once reject this as quackery, enquiring into which is beneath their dignity, we beg to submit the following quotation:—"Having paid some attention to the proceedings of empirics, foreign and indigenous, regular and irregular, we venture to say, that we may sometimes be taught by them useful lessons; and we ought not to decline assistance even from such sources." This was written by a physician of the highest reputation of his day; who to a scientific knowledge added great practical skill, gained by watchful, long, and very successful consultation-practice in a city famed for the celebrity of its medical men—by the late Dr. Cheyne, of Dublin.

Vincent Preissnitz, the founder of what is called the Hydropathic System, is one of those self-educated men who occasionally become notorious by their own unaided efforts in a path of their own selection; men who know not such a word as "impossible," and for whom things which are hinderances to others, are among the means of their success. Reserved, silent, a man of the fewest words, who promises nothing; irritable and impatient at opposition; with a strong will, unyielding firmness, and untiring perseverance in carrying out his plans; and withal thoughtful and watchful, capable of adapting his means to the individual circumstances, and fruitful in expedients under unexpected difficulties: such is the character which he shares with many of those whom the world willingly worships; or, rather, to whom small sections of it everywhere submit as their guides or rulers. The unbounded confidence which such men have in themselves seems to be willingly believed in by almost all who are directly subjected to their influence.

Preissnitz's father was a small farmer in the mountains of Silesia, who brought him up to the severe manual labours of an agricultural peasant. His inclination to his present very different condition seemed to have depended on an old man, who was accustomed to use the water-cure in diseases of cattle; and he taught the art he himself possessed to Preissnitz. He applied the same remedies to his sick neighbours, and was confirmed in his views (as doctors often are,) by the good he gained by treating with water dressings a severe injury which he himself experienced. The success of his own case was also the means of spreading his fame. He is now forty-three years old; his reputation has gradually increased, so that within the last dozen years between 7000 and 8000 persons have submitted to his treatment under his own eye.

Besides the numerical method there are, we know, two other processes of proving a medical man's success—by the money the favorite accumulates, and the "great" people who put their faith in him, and submit their "porcelain clay" to his care. By both of these tests Preissnitz may be tried, and his celebrity strictly proved. "At present, in 1841,"

says Mr. Claridge, "there are under his treatment an archduchess, ten princes and princesses, *at least* 100 counts and barons, military men of all grades, several medical men, professors, advocates, &c., in all about 500!" (p. 69.) And besides this high patronage, he has accumulated solid pudding to the amount of £50,000; not from the accumulation of guinea fees, or journeys at a guinea a mile, or occasional cheques of £1000 in nightcaps thrown at the surgeon's head, but from fees ranging from the minimum of four shillings a week to the maximum of double that small sum, and from the profit arising from his great boarding-house, where his patients are fed for eight shillings a week, and lodged for four shillings more. For, notwithstanding his celebrity among the celebrated —in spite of a list of patients which reminds one of the court circular on the day after a drawing-room, Preissnitz receives several hundred patients into his own house, which has been built for the purpose, and his wife, "who," says M. Gross, "is pretty and fair, very natural, but very clever," is perfectly acquainted with domestic economy, and alone manages the entire household. It speaks well for him that he retains much of the simplicity of his former station, both in habits and manners— rising very early, temperate, and a water-drinker, and still keeping up the custom of the poor in that part of Europe, of kissing the hands of their superiors (especially of all ladies,) on entering and leaving the room.

The information we have of his treatment is not from his own writings, as, from want of education, he is probably no writer; nor from his own mouth, as he is no talker or explainer of his plans and views. The number of his patients must prevent his talking much with each; and although his practical ability may be considerable, yet his ignorance of the science of medicine, and, probably an inability to explain clearly the rules which guide him, may make him pursue what is certainly his safest course—that of not explaining anything. He is said to have an aversion to medical men: this is not unlikely, as he must have met with opposition from them; and irregular workmen of all crafts look suspiciously on those into whose legal province they are intruding. What means we have of judging of Preissnitz is from the works of medical men and of others who have visited him as patients. The two English works at the head of this article are those which we shall make use of. One is written by Mr. Claridge, a non-professional gentleman, who was the first to introduce Preissnitz to the British public in a separate book. After suffering from rheumatism for many years, which was not relieved by the usual remedies, or the regular physicians to whom he submitted himself, he went to Graeffenberg, and was cured. Active-minded, acute, and observant, he has described the means of cure he witnessed, and has translated the opinions which many German physicians have published after visiting the same establishment. Like many intelligent people who are ignorant of the practice of medicine, especially if they have suffered from a disease which has baffled the ordinary remedies, Mr. Claridge sees clearly enough that the medical art is imperfect, and then hastily arrives at the conclusion that there ought to be a perfect system of cure, that there must be means to remove without fail the actual cause of the disease. Such persons, with their scanty stock of knowledge and their enormous self-conceit, are the easiest prey to quacks. The homœopath horrifies them by describing the consequences of bleeding and purging, remedies, as he says, directed to effects only, whilst he adds, "this little globule goes at once to the cause, and destroys that without any injury to the constitution," and they believe him. The advertisements of our newspapers show us daily that both regular and irregular quacks—from doctors with diplomas who advertise their books, to the chemist who trades in his peculiar pill—adopt the same principle of delusion; strongly setting forth some obvious defect in the medical art, and announcing unscrupulously that the new remedy or new plan supplies infallibly the great deficiency. Mr. Claridge has evidently no doubt at all but that all diseases depend on some material poison, some materies morbi, and that this is to be eradicated, not by drugs, but by water, producing perspiration and eruptions, boils and abscesses, which contain the true cause of the disease itself. But although we do not seek in Mr. Claridge's book for either just or comprehensive views of the nature or treatment of diseases generally, or for a fair estimate of the advantages of the system he upholds, compared with other plans, yet, from his having submitted to the treatment, he is fully competent to describe the mechanical plans adopted, and the regimen to which he was subjected. He is an enthusiastic and undoubting believer, and seems sincerely desirous to communicate to others the knowledge of means which in his case have proved so serviceable. And on this account his book is likely to be more convincing to others than the second pamphlet, the work of a medical man, who (as we see by the daily advertisements,) uses it as the means of acquainting the English public that they may be cured in the same way at home. This book is written in too jocose and flippant a style to be of much weight. Quotations from Don Juan do not enhance the value of a medical book. The writer is also one of those practitioners who never lost a case of scarlet fever, and who reflect on the treatment when a fatal epidemic occurs—a sure proof that he has seen very little practice. On the whole, therefore, we give the preference to Mr. Claridge's work, as one written with more earnestness and singleness of purpose.

The channel by which Preissnitz cures, or attempts to cure disease is through the medium of the skin; he endeavours to produce perspiration or cutaneous eruptions, boils or abscesses. For this purpose he employs water as an external application and as a beverage, together with exposure to air, exercise, and strict abstinence from stimulating drinks. He does not indiscriminately attempt all cases. He refuses to treat patients with diseases in his opinion incurable, such as consumption, organic defects, or where the *vis vitæ* is manifestly exhausted; but we should gather from the books before us that he subjects all diseases which are curable by medicine, to his plan. But although using one plan, he modifies that very considerably, according to the constitution of the patient, his strength or weakness, and the seat of the disease. And in thus accommodating his means to his ends he is said to show much judgment. In theory he is exclusively a humoral pathologist, regarding all diseases as dependent upon a morbid state of the fluids, and that the cure depends on an evacuation of the diseased matter in a visible form, in sweat or pus, and that this evacuation is preceded by a febrile state of the system—the body struggling with the disease and throwing it out. This crisis he seeks to produce.

Graeffenberg, where Preissnitz lives, is a small hamlet, half way up one of the mountains (called Sudates) of Silesia, in Austria. He has accommodation in his own houses for about 200 patients, and the villagers can lodge 150 more. The fastidious would not be suited. The cottages seem equal to the lowest dwellings of our agricultural poor. "What a bad lodging!" exclaims M. Gross. "Our servants would not put up with it. My landlord conducted me by a narrow staircase, almost perpendicular, placed at the low and dirty entrance of the house, to a very small room, so low that I could not stand upright in it. I constantly ran the risk of knocking my head against the beams of the ceiling." Even those who are fortunate enough to get a room in Preissnitz's own establishment (and this is only done by coming very early in the season, or writing to hire one beforehand,) have to put up with much. The bed-room is equal to a soldier's barrack-room. A bedstead and a straw mattress, a deal table, two chairs, a chest of drawers, a large basin, a decanter and a glass are the sole contents. The whole place, too, is permeated with a smell, compounded of the cows which are kept in stables beneath the house, of cabinets d'aisance upon the staircase, and of the kitchen beneath the common room, which opens into it by a trap door, through which the cooked food, as well as the various odours find entrance. The less comfort there is within doors, (thinks Preissnitz,) the less time will my patients spend at home; and the coarser the fare, the less danger will there be of their bodies being too highly nourished by it. Three miles from Graeffenberg is the town of Freiwalden, where those are accommodated who cannot find room nearer, and where there are better lodgings. Those who wish to put themselves under Preissnitz's care are recommended to write their case to him in French or in German. as he will not undertake those patients whom he considers to be incurable.

If the patient is of a sufficiently vigorous constitution, he is submitted to a sweating process—one of considerable originality. At four in the morning, the servant throws off the patient's bedclothes, and wraps his body closely in a blanket, covering this with a small feather or down bed, placing a counterpane over that, and tucking all tightly in. In a space of time varying from half an hour to two or three hours, he begins to sweat; the windows are opened, he is allowed to drink cold water, and the sweating is kept up from half an hour to two hours. Then the bed and counterpane are removed, the face and neck wetted with cold water, and the patient (still covered with the blanket,) puts on straw shoes and walks to a cold bath in an adjoining room. He again wets his face, neck, and chest with the cold water, and then, wet with perspiration, plunges into the cold bath, and remains there from two to eight minutes. When it agrees, reaction follows, and the skin becomes red. He dresses, walks, and then breakfasts. This is repeated daily, or every second or third day. Or instead of the whole-bath, a douche or a half-bath may be substituted. This is the occupation of the early morning: douche and local baths and wet compresses employ much of the day. For those who are too weak for this process the *wet sheet* is used. A sheet is well wrung out of cold water, it is then spread upon a blanket, and wrapped round the patient, and upon this is placed a feather bed, as in the former process; afterwards a cold or half-bath. Preissnitz treats fever and febrile diseases, such as scarlatina, smallpox, and the like, with this wet sheet. It is also used as a portable bath. On the patient's getting out of bed, a wet sheet is thrown over him; he well rubs the front and the attendant the back of his body for several minutes; a dry sheet is then substituted, and the same friction repeated until he is dried. It is said to be a convenient bath when travelling, relieving fatigue, and rendering the skin less sensitive to cold. Very delicate persons are washed all over, water being poured over them whilst the attendant rubs the whole body and the affected part with the hand. Where the whole bath is too much for the patient's strength, and generally as a preparatory step to the sweating process, a *half-bath* is used: the water being from three to six inches deep, and not below 60°. Local half-baths are much employed. A *sitting-bath*, in which a person sits as in a hip-bath, in water a few inches deep; a *foot-bath*, with two or three inches of water; a *head-bath*, the patient lying with the back of his head in a basin. There are local baths for all parts—eye, elbow, leg, and finger baths. The *douche* is not merely used locally, but generally. A mountain spring is carried through pipes into rude huts, so as to fall from ten, twelve, eighteen, and twenty feet, in a stream the size of the wrist. The patient (perhaps after sweating,) first washes his face, head, and chest with his hands; then he allows the stream to fall slanting upon his shoulders, hips, and loins; then upon his head, guarding it with his hands held over it roofwise; and then upon the weakened or paralysed part, if such there is. He dresses in a similar hut close by; and generally much enjoys this part of the treatment. The time varies from three to fifteen minutes. In those benefited by it the skin becomes of a rosy red; it gives tone to the muscles, and increased activity. If it produces feverish symptoms, it is discontinued. *Wet compresses* are much employed, not merely to produce cold by evaporation, but as we use "water dressings," only instead of oiled silk, the wet compress is covered with a dry linen compress, and changed when dry. But besides employing these for all kinds of wounds, ulcers, &c., and nothing else, Preissnitz orders them for every sort of local disease, such as come within the province of the physician as well as the surgeon. Those whose throats or chests are affected wear them around their necks and chests at night; the gouty and rheumatic have their legs incased with wet clothes at night; and those affected with any abdominal disorder, such as indigestion, feeble digestion, constipation, diarrhœa, colic, and the like, wear a wet towel round their bowels during the day. A towel three yards long and one foot wide, two thirds of which are wetted, is thus applied: the wet part is wound round the belly, and the dry part covers it, strings being attached to the dry end.

But besides the external application of water, Preissnitz enforces a general regimen.

He directs his patients to drink as much cold, pure, spring water as they possibly can swallow without feeling a painful distension of the stomach. Before breakfast is the time at which most water can be drunk, but it is to be taken at meals and between meals also. Habit does much: in a short time from twenty to thirty glasses can be drunk daily. He allows neither beer, wine, nor spirits; no tea, nor coffee, nor any hot fluid. He allows his patients to eat at will of coarse foods; he does not limit their quantity, nor is he careful as to quality, as his table is supplied with (what we consider) very indigestible food, such as pork, veal, ducks, geese, cucumbers, and all kinds of pastry. All mental occupation is forbidden, and much exercise, with constant exposure to the open air, required.

The breakfast consists of cold milk, brown bread and butter, and wild strawberries, which grow abundantly there from May to October; the evening meal at seven p. m. is the same. The dinner at one o'clock consists of soup with beef boiled in it, followed by pork, veal, or mutton alternately, fowls, ducks, geese, salads, cucumbers, and preserves, and all kinds of pastry. Vegetables—except cabbages and sour-krout—are very scarce, but are permitted. The patients at the tâble d'hôte—where upwards of 200 are congregated—are described as eating indiscriminately great quantities, amidst much noise and merriment; so that the new-comer finds it difficult to believe that so many healthy-looking, happy, mirthful people are very sick. Mr. Claridge's own case will exemplify the application of these means.

At four in the morning he was wrapped in a blanket, covered with a bed, and kept in a profuse perspiration for an hour, and then went into a cold bath for three minutes. He dressed, drank cold water abundantly, and walked until breakfast. At ten a douche-bath at some distance; and between this and dinner, at one o'clock, he took a sitting-bath and a foot-bath, remaining in each a quarter of an hour. Another douche at four in the afternoon, a sitting and a foot-bath again at seven, and to bed at half-past nine, sleeping with both legs wrapped up in wet clothes. During the intervals he walked vigorously as much as ten miles every day; and he says he was in more robust health than he had ever known. His disease was rheumatism of many years' standing. As he seems to have been subjected to the most rigorous application of water, such as a daily sweating and the douche twice every day, we conclude that he must have a robust and vigorous constitution; and the enthusiasm in which he talks of the effects of abstinence from stimulants and of cold ablution, indicates one to whom these very obvious means of health are novelties. This treatment was persevered in for three months.

The information we have as to the application of these means to the various diseases is by no means satisfactory. The pamphlet by Dr. Wilson is on this point extremely scanty; and Mr. Claridge's want of knowledge of medicine and enthusiastic zeal incapacitate him very much as a witness, although his attention to the subject makes him the best witness we have. The translations which he gives from the works of foreign physicians do not indicate that the writers have sufficiently studied for any length of time the practical application of the treatment. Gout and rheumatism were the diseases which Preissnitz first attempted to cure; but it seems that individuals with all kinds of chronic diseases, such as resort very commonly abroad to mineral springs and baths, put themselves under his care: and that when any of these whilst residing with him are attacked with acute inflammations, or with fevers, or eruptive diseases, that they are subjected to similar means of cure. Many of these chronic invalids are affected with diseases of their digestive organs and their sequelæ, and when brought about by full living, drinking much beer, &c., incessant smoking, with the sedentary habits, confinement to the house, deficiency of pure air, and uncleanliness of skin and linen which such sensuality supposes, the beneficial effects of so contrary a system are not surprising. Those who are of weakly or delicate constitutions, whatever may be the local disease, are evidently not subjected by Preissnitz to his heroic treatment. Frequent cold ablutions, the body being at the same time rubbed by the hand of an attendant, and *half*-baths, in which there is but a small quantity of water, which soon becomes of the temperature of the body, is substituted for the sweating process; and it seems that Preissnitz, as he has more experience, is more cautious in the application of his remedies. A man himself of the strong, much-enduring frame of a peasant, who began the practice of medicine upon horses, cows, and dogs, and passed from animals to his own poorer countrymen, living, like himself, the simple hardy life of mountaineers, must require some experience to teach him how wofully civilization and luxury and refinement impair the native vigour of the constitution, and render practices hazardous in one case which in the other were at least harmless. Scrofula is mentioned as successfully treated. The external application of water as a general tonic, as well as an application to enlarged glands, ulcers, &c., together with the mountain air, exercise, and milk diet, may be in many cases beneficial; but as strumous patients are usually of feeble constitution, they are not probably submitted to the most active treatment. Hysteria and hypochondriasis are subjected to the treatment according to the strength or weakness of the constitution. Patients are not placed in the bath during the attack of epilepsy, or whilst in a nervous convulsion. Diarrhœa and cholera are treated with sitting-baths for half an hour three or four times a day; cold water in abundance, as drink and as clysters, and a wet compress over the abdomen. After the bath the patient is put in bed to encourage perspiration.

In *congestions in the head* the sweating process is omitted: but sitting-baths with cold, wet bandages to the head, with abstinence from all stimulants and excitements, mental and physical.

In *habitually cold feet,* besides abundant exercise, cold foot-baths twice daily for fourteen to twenty minutes; and wet compresses at night. The same treatment for fetid perspiration from the feet.

*Chilblains* are covered with wet compresses.

In *headaches*—especially gouty headaches—and for deafness the head-bath is used. The patient lies on his back, with the back of his head in a basin of water for a quarter of an hour, then he turns on one side, then on the other, and then on his back—thus keeping his head in the water for an hour. We are not surprised to hear that this often produces abscess in the ear: Preissnitz reckons such a consequence critical and beneficial; but to us it seems a very hazardous and rash proceeding, and that the abscess might destroy the ear entirely.

*Toothach* is said to be relieved thus: either by a cold foot-bath, or by the patient's keeping tepid water in his mouth and constantly changing it as soon as it gets warm, whilst he puts his hands in a basin of cold water and rubs his face and the back of his ears violently with them, *until the pain ceases.*

For *habitual costiveness* two or three cold clysters are given daily; much cold water is drunk; a wet compress is worn over the belly; cold food, and plenty of fruit. If it has lasted for years, the sitting and foot bath and the douche.

For a *common cold* perspiration in the wet sheet, or the half-bath is said to be curative. The same treatment for gripes.

For *sprains and stiff joints* cold local baths and wet compresses; for *fractures* wet bandages; for *sores*, *ulcers*, *wounds*, *bruises*, wet compresses.

In *fevers* and *eruptive fevers*, as *smallpox*, *measles*, *scarlatina*, &c. the patient is wrapped in a wet sheet, which is often renewed, and this is covered with bedclothing; when perspiration breaks out, a half-bath at 61°.

In *inflammation of the lungs and abdomen* the patient is placed in a sitting-bath at first, and the legs, which are out of the water, are well rubbed. Cold, wet, evaporating cloths are placed over the chest or abdomen, and frequently renewed, and the body is wrapped up. This is said to produce warmth, and then the wet sheet is used.

In *intermittents* the patient is placed in a *half-bath* and rubbed during the cold stage, and he is made to drink plentifully until he vomits. Wet compress over the belly.

*Itch*, *piles*, *diseases from syphilis and from mercury* are subjected to the full treatment.

Any estimation of the value of hydropathy as a means of curing disease must at present be imperfect, as the medical evidence (from causes previously mentioned,) is very scanty. There is little more indeed than the bare fact that several hundred persons annually submit themselves to this treatment for several weeks, or even months, together, without medicines of any kind. And it appears that the invalids are such as usually resort to mineral spas abroad, with chronic diseases of all sorts, all of which unless they have arrived at a stage manifestly incurable, are subjected to the same means, modified according to the constitution of the patient, or the locality of his complaint. It is but just to admit the improbability of as many as 1400 patients in one year, and for several years in succession, submitting to such a course of treatment unless it were in many instances beneficial. It may be said, that the same argument would prove the benefit of Morison's pills, the sale of which is said to have been for many years most considerable. The difference between the two plans of treatment, however, is very great as regards their effects on the body. Purging, and aperients relieve many diseases and disordered conditions of the body during their administration, and it is not discovered until some time afterwards that the constant use of such stimulating medicines has materially injured the constitution. But in hydropathy the injury, if any, must be felt at the time. If a patient has strength enough to undergo these hardening processes, we cannot imagine any subsequent mischief. It is then but fair to conclude that if the system had been a dangerous one, as the injurious effects would have been at once apparent, the success which has attended it would not have followed, and that Preissnitz has performed experiments of the effect of pure cold water upon the body on so large a scale, and for so long a period of time, as to merit our inquiry and attention.

Preissnitz's remedies are of three kinds:

1. The administration of the cold-bath when the body is in a profuse sweat, not from exercise, but from warm clothing. The principle that as a rule there is no danger in applying cold to the surface when in a state of perspiration, unless the body is at the same time fatigued or exhausted, is known and acknowledged. The Russian vapour baths and the mode in which the Indian treats fever, prove medically what every one who has seen schoolboys bathing in hot weather, or Neapolitans drinking iced water must have felt to be true—that cold may be applied externally and internally with impunity, even if the skin is profusely perspiring from mere external heat; whilst sudden deaths in our harvest-fields and the injury even from local application of cold water after heat produced by fatiguing exercise are an opposite class of facts. Preissnitz is original, not in discovering this principle, but in its application. He produces perspiration by warmly covering the body, and then subjects it to cold. By this means it appears that great action of the skin is produced. The free sweating in bed, promoted by large quantities of cold drink, proves that his diaphoretic remedy is a vigorous one; and the effect of the cold bath afterwards is, according to those who have tried it, to make the skin red and warm; thus increasing, obviously, the whole capillary circulation. These means of curing diseases are therefore powerful ones. Practically we must all own that we can cure very few diseases without producing evacuations. The humoral pathologists of former days conceived that in this way the actual materies morbi—"the humour"—was got rid of. Since John Hunter's time we talk learnedly of a new action being set up to supersede the old and diseased one. But whatever may be the theory, it cannot be doubted that diaphoresis produced every morning for several weeks must have a considerable effect on the economy; and if it can be produced without debilitating the body, but by means which actually strengthen it, it is a judicious mode of attempting the cure of many chronic diseases. We have in this country a somewhat analagous process in training. When the greatest muscular strength is necessary, as in men preparing for boxing or pedestrianism and for race-horses, regular sweating is thought essential to the process; so that in practice it is found to be a necessary part of a tonic regimen. But in addition to ordinary perspiration and reaction subsequently from cold, the treatment, when persevered in, produces a general febrile disturbance of the whole system, sometimes trivial and sometimes severe, and often attended with skin-eruptions, boils, and abscesses. In this country we are extremely unwilling to admit the doctrine of crises: we take matters so entirely into our hands, and use the bodily machine as if it were a passive piece of mechanism that we have to clean and regulate, that we are almost sceptics as to the influence which Nature herself exerts in curative processes. But it is with due consideration that almost all physicians who watch the effects of mineral waters of all kinds upon the body are agreed as to one point —that the waters or baths act by producing a febrile disturbance in the system, followed by some visible excretion or new action. Bordeu, who studied for years the action of the *simple thermal* waters of the Pyrenees; Marcard, who observed the effects of the *Chalybeate* of Pyrmont; Heidler, who is still the resident physician at Marienbad, where the most active spring is a *saline aperient;* and Bertrand, who with personal attendance watches the action of the *hot alkaline* baths of Mont d'Or—

all men of practical sagacity, observation, and judgment—besides a host of others, agree in this point. Preissnitz, also lays much stress on this disturbance which his cold water applied so constantly is likely enough to produce; and he regards it as indicative of cure.

2. But besides sweating, followed by a cold bath, perspiration is produced by the application of a wet sheet to the whole body, and by wet compresses to various parts of the body. A similar plan was common in the northern parts of England a century and a half ago. Children at a very early age with rickets were taken to the wells of St. Bede, Honwick, or St. Mungo, "which are extream cold springs," and thus treated. "Some dip them," says Dr. Ellison, in a letter to Sir John Floyer, dated 1701, "twice and thrice over head and ears, with their shifts and nightcaps on, in the evening, for a fortnight or longer. Others dip them no further than the neck. Others, where the well is not capacious enough, content themselves to put the children in a tub of water gathered from the spring, and dash the water upon them over head and ears. All which immersions are to be despatched as quickly as may be, so that the child may not continue any longer in the water than is necessary; that is, till his body, and shirt, and night-cap be thoroughly wet. Others, out of tenderness to the child, or in regard to the child's weakness, content themselves with dipping only the shirt and nightcap in water, and put them on wet upon him. As soon as the children are dipped, they, *with their wet clothes on*, are wrapt up in warm blankets over their head and whole body, and put immediately to bed, which instantly puts them into a violent sweat. In this condition they lie all night, till towards morning the clothes are taken off by degrees, that so they may cool gradually; and in the morning they have dry shirts and head-clothes put on."* The writer adds that he never heard of any children who died of dipping, and of few or none but found great benefit. There are frequent indications in books of the same period of "water dressing" being applied by the poorer people who frequented springs in the same wholesale way. "There is," says Sir John Floyer, "a dangerous practice at Willowbridge, (a cold spring,) of which I have heard some patients complain: they wear the wet shirts in which they bathed all day afterwards, by which some were over-chilled; but I have heard others, that were more strong, who bore that practice without any injury, as they informed me." Saunders, in his work on Mineral Waters, mentions the practice of the country-people at some of the springs of very pure or tepid water was, to keep clothes, constantly wet, to the diseased or weakened part. The substitution of water-dressings for poultices, introduced by Dr. Macartney, need hardly be alluded to as a great improvement in the treatment of wounds and injuries. Preissnitz has carried it still further, using water-dressings for all local diseases which come under the province of the physician, as well as to all external ulcers, wounds, or bruises. He does not use oiled silk, but covers a wet compress with a dry one. The half-baths are a peculiar part of his system: sitting in a tub, the water of which is but six inches deep, or using a foot-bath, with the water only just covering the toes, for a quarter or half an hour, or even more, at a time, and repeating this more than once during the day, are processes to which he subjects his patients in order to produce a determination of blood to the part immersed. The shallow water soon becomes

* Floyer's History of Cold Bathing. Letter Fourth, p. 129.

of the temperature of the part which is immersed, and the patient does not seem to be chilled by it. The application of the douche to every part of the body in succession is an improvement on its mere local application, and it must act as a powerful tonic to those who are strong enough to bear it. The application of wet sheets, with external warmth in cases of fever, seems to be worthy of a trial in such cases as are likely to be benefited by perspiration, where the skin is hot and dry and the circulation vigorous.

3. *The frequent application of cold water.* The effect of cold water in hardening the skin and rendering it less sensitive and less liable to be affected by changes of temperature, is well known. By cold bathing alone, if we consult enthusiastic admirers of it, we shall learn that Preissnitz has been anticipated in the cure of gout, rheumatism, all kinds of neuralgias, hysteria and hypochondriasis, fevers, agues, asthma, and a variety of chronic diseases.*

Hydropathy would teach us one useful lesson if it would direct our attention more closely to the beneficial effects of this common remedy. There is no tonic more efficacious, and no single means more likely to decrease that sensibility of the skin to external influences, from which so many invalids suffer from constant colds and their sequelæ, than the outward application of cold water; and where a cold bath cannot be provided, or a shower-bath, a sponge and a large tub is an excellent substitute with which the body should be rapidly wetted on getting out of bed every morning. Foreigners reproach and satirise us for copying, to our own great inconvenience, the habits of the highest classes. There is one of their habits which cannot descend too low—extreme cleanliness from the plentiful use of water. Now that all external distinctions of rank, from clothing or decorations, have gone into disuse, there seems but one external mark left, that of cleanliness; the degrees of which, to a minute observer, are criterions of rank approaching the infallible.

With regard to that very important part of Preissnitz's treatment, his regimen,—it is on the whole so good that many of his cures must be alone attributable to it. Bathing ensures extreme cleanliness of skin; early hours, abstinence from all stimulants, both physical and moral, full exercise in a clear mountain air, a plain, simple, and coarse diet, such as exercise and air alone would enable invalids to enjoy or to digest with the appetites "of hungry workmen,"—is a mode of living which is very rational. All studious exercise of the mind is forbidden, and indeed there must be so much dressing and undressing, so much dipping and dashing and paddling and splashing, and rubbing and scrubbing—as to furnish full employment. The unlimited quantity of food permitted is objected to by both writers; perhaps Preissnitz has discovered the uselessness of limiting the *quantity* of food where air and exercise produce the appetite of a "workman," and is content in setting before his patients coarse and plentiful viands. The abundance of pure cold water which is taken must supply copiously the fluid for perspiration and thus contribute to the cure. All those patients who have been previously accustomed to wine, spirits, or beer, which, as a general rule, must be hurtful in all diseased conditions of body, would, of course, feel a marked improvement

* See History of Cold Bathing, by Sir John Floyer, Knight, and Dr. Baynard, Ryan on Asthma, &c.

merely from the substitution of a milder liquid; whilst those who are disposed to eat more than is useful, must find less room when parts of their stomachs are occupied with this less substantial article of their host's dietary.

This question may perchance be put to us,—Would you recommend me to try Hydropathy? And in such cases the inquirer has usually almost made up his mind to try it, and asks for confirmation. The answer would be, that Hydropathy is no amusement; by no means similar to a trip to one of the German Brunnens; but that much inconvenience must in every way be submitted to. If, however, it is a case of confirmed chronic disease, not incapacitating the patient from the long journey, not curable by ordinary methods, and not glaringly incurable, much seems to depend on the constitutional strength of the individual. If there is still vigour of constitution there would be less doubt in approving of the journey. If the disease was the effect of intemperate habits, and a journey to Graeffenberg alone was likely to cure these, by all means let your patient go. If he will not take sufficient exercise, if he is scanty in external ablutions, and if his habits are not to be broken through at home, and on these his indisposition depends, even a journey to Silesia will not be too laborious a means of benefit. But if hydropathy is to be tried, we should recommend the invalid who is bent upon it to go to Preissnitz himself. Setting aside the advantages of his local habitation and his name, we would trust more to his strong will and bold and energetic ignorance than to the calculated refinements of his would-be scientific followers. We have faith also in that "art unteachable, untaught"—that *medical tact,* which seems native to the man, and which, had his station in life and his education been different, might have made him, instead of the inventor of the empirical water-cure, the all-admired Hippocrates or Sydenham of these latter days.

---

